# Resistance Switching Effect of Memory Device Based on All-Inorganic Cspbbri_2_ Perovskite

**DOI:** 10.3390/ma14216629

**Published:** 2021-11-03

**Authors:** Wang Ke, Xiaoting Yang, Tongyu Liu

**Affiliations:** 1 Science and Technology on Electro-Optical Information Security Control Laboratory, Tianjin 300308, China; liu_tongyu@163.com; 2 School of Physics, Beihang University, Beijing 100191, China; yangxiaoting@buaa.edu.cn

**Keywords:** CsPbBrI_2_ perovskite, non-volatile, resistance switching effect, memory device

## Abstract

In this study, the CsPbBrI_2_ perovskite film was prepared by the preparation of the sol-gel and the spin-coating method, and the cubic lattice was stabilized by introducing Br^+^ into the CsPbI_3_ film, which solved the problem of instability of the traditional perovskite phase. Based on the CsPbBrI_2_ perovskite film, the Ag/CsPbBrI_2_/ITO memory device with a resistance switching effect was prepared. The morphology and phase compositions of the film were analyzed by scanning electron microscope and X-ray diffraction. The non-volatile and repeatable resistance switching effect of the Ag/CsPbBrI_2_/ITO memory device was measured under open-air conditions. The experimental results show that the surface of the CsPbBrI_2_ perovskite film is uniform and dense, and the Ag/CsPbBrI_2_/ITO memory device has an order of magnitude resistance-on-off ratio after 500 cycles of cyclic voltage. This study shows that Ag/CsPbBrI_2_/ITO memory devices based on CsPbBrI_2_ perovskite films have potential applications in the field of non-volatile memory devices. At the same time, the transient properties of the CsPbBrI_2_ film that can quickly dissolve in deionized water make it potentially useful in short-period data storage units and implantable electronic devices with human or environmental sensors.

## 1. Introduction

Due to the rapid development of semiconductor technology, transient electronic memory devices have reached their limits of scale. The research of new memory devices is very urgent for the high cost and power limitations of the current transient memory devices. There are four existing memory types, such as Ferroelectric Random-Access Memory (FeRAM), Magnetoresistive Random-Access Memory (MRAM), Phase Change Random-Access Memory (PRAM), Resistance Random-Access Memory (ReRAM) and charge trapping memory devices of two-dimensional materials. Resistive random-access memory is a new type of random-access memory device based on the resistance switching effect. It has outstanding superiority compared with traditional random-access memory due to its scalability, low power consumption, fast switching speed, durability and long data retention characteristics [1,2,3,4,5,6]. Resistance Random-Access Memory (ReRAM) is composed of a sandwich structure in which the upper electrode is usually made of metal oxide or alloy, and the lower electrode is usually made of oxide or carbon-based materials. According to the current studies, the middle active layer is mainly binary oxide, perovskite oxide, van der Waals material, biomass material, etc. [7,8,9,10]. As an intermediate active material for a new generation of resistive random-access memory, organic–inorganic halide perovskite materials show a more remarkable resistance switching effect than traditional active materials due to their simple preparation process and the unique current-voltage hysteresis property caused by rapid ion migration and defects [11,12,13,14,15,16]. However, the application of the organic–inorganic halide perovskite materials in the resistance switching memory devices is limited due to the hygroscopicity and relatively poor thermal stability of organic cations [17,18,19,20]. According to the reported literature, the organic cations in the organic–inorganic halide perovskite materials can be replaced by inorganic cations such as Cs to improve the stability of the material, which shows that the all-inorganic halide perovskite material (CsPbX_3_, X = Cl, Br, and I) as an intermediate active material of resistance random-access memory devices has potential advantages [21,22,23,24,25,26]. Because of the more stable perovskite phase compared with the CsPbI_3_ halide perovskite, the current research mainly focuses on CsPbCl_3_ and CsPbBr_3_. Therefore, it is very necessary and meaningful to solve the stability of the CsPbI_3_ perovskite phase and prepare a resistance switch random-access memory based on the CsPbI_3_ halide perovskite [27,28,29,30].

In this work, the CsPbBrI_2_ is prepared on ITO glass by spin-coating using a smaller Br^+^ cation to partially substitute Pb^2+^ into the CsPbI_3_ perovskite. A stable film is obtained after a low-temperature annealing process. Finally, a resistance random-access memory of Ag/CsPbBrI_2_/ITO structure is obtained with the silver prepared on the upper layer of the film by ion sputtering as the top electrode. An order of magnitude resistance-on-off ratio is obtained by scanning the memory device with a cyclic voltage, which shows that the device has a non-volatile, reliable and stable resistance switching effect. In particular, in order to meet the needs of transient information storage, there is a great need to develop transient resistance switching devices. As we all know, the CsPbBr_3_ is the most commonly studied transient resistance switching memory device. In our research, we found that the CsPbBrI_2_ film is completely dissolved in deionized water within 3s, which exhibits a potential application in transient resistance switching memory devices with transient properties.

## 2. Experimental Section

### 2.1. Preparation of CsPbBrI_2_ Perovskite Precursor Solution

One mmol mixture of CsBr/PbI/CsI/ was fully dissolved in 1.5 mL of mixed solvent, which is prepared by uniformly mixing DMSO (dimethyl sulfoxide) and DMF (N, N dimethylformamide) at a volume ratio of 2:1. Afterward, the mixed solution was uniformly stirred under magnetic stirring for one hour to obtain a CsPbBrI_2_ perovskite precursor solution at room temperature [31,32].

### 2.2. Ag/CsPbBrI2/ITO Memory Device Fabrication

The ITO glass was ultrasonically cleaned with detergent ionized water and acetone for 5 min and then placed in a drying box for drying in a nitrogen environment. Subsequently, the CsPbBrI_2_ perovskite precursor solution was spin-coated on the dried ITO glass at the speed of 4500 r/h for 30 s. The annealing treatment process of obtained CsPbBrI_2_ perovskite film, a uniform film, was carried out at 250 °C for 1 h, and then a CsPbBrI_2_ perovskite film was prepared. Afterward, the Ag electrode with the scale of 1 mm × 1 mm was deposited on the CsPbBrI_2_ perovskite film by direct current magnetron sputtering using a shadow mask to complete the Ag/CsPbBrI_2_/ITO resistance switch memory device [33,34].

### 2.3. Analysis and Characterization of Device 

The field emission scanning electron microscope was used to analyze the surface morphology of the CsPbBrI_2_ perovskite film and the cross-sectional morphology of the Ag/CsPbBrI_2_/ITO device. The X-ray diffraction pattern was used to analyze the phase compositions and crystal structure of the CsPbBrI_2_ perovskite film. The I-V characteristics of the device were measured by using a semiconductor parameter analyzer at room temperature to test and analyze the resistance switching effect of the Ag/CsPbBrI_2_/ITO resistive random memory device. 

## 3. Results and Discussion

**Figure 1:**
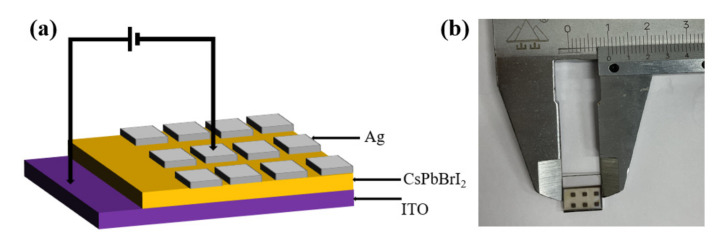
The schematic diagram of the Ag/CsPbBrI_2_/ITO resistive random memory device (**a**) and the original optical image of the final device (**b**).

The schematic diagram of the Ag/CsPbBrI_2_/ITO resistive random memory device (a) and the optical image of the final device (b) are shown in Figure 1. The typical sandwich structure of the device can be seen from Figure 1a, in which the CsPbBrI_2_ film is used as the middle active layer, and Ag and ITO glass are respectively used as the upper and lower electrodes. By applying a voltage between the two electrodes, a resistance random-access memory is formed. 

**Figure 2:**
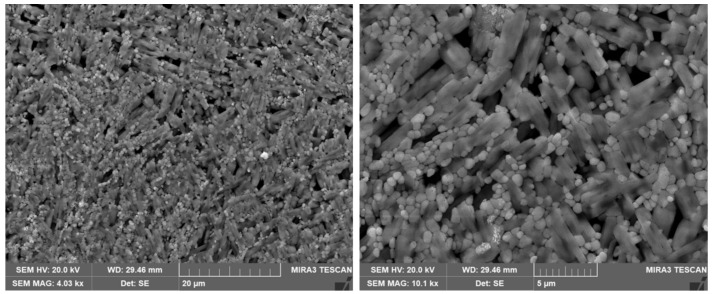
The scanning electron microscope surface morphology of the CsPbBrI_2_ perovskite film.

**Figure 3:**
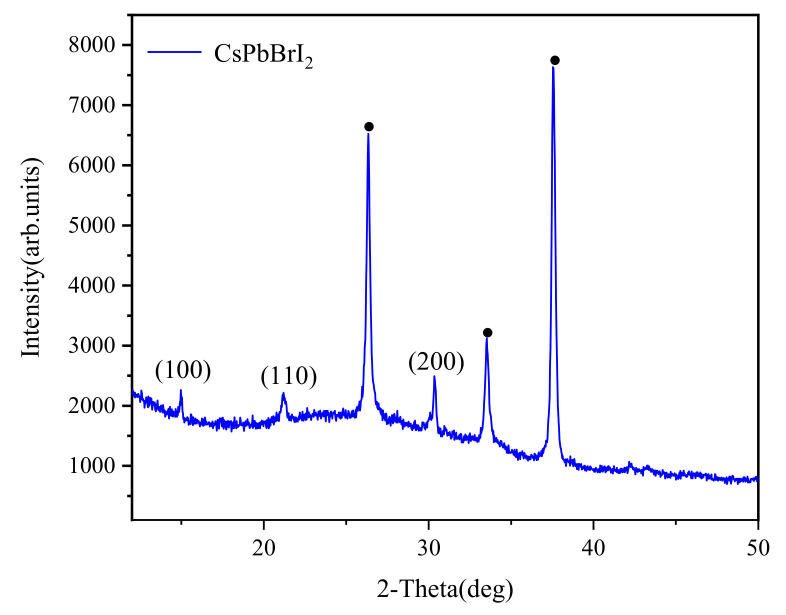
The X-ray diffraction pattern of CsPbBrI_2_ perovskite film.

Figure 2 shows the scanning electron microscope surface morphology of the CsPbBrI_2_ perovskite film. It can be seen from Figure 2 that the particles on the film surface are uniformly distributed, it shows that the CsPbBrI_2_ perovskite film prepared by this experimental preparation method has good stability with the thickness of 1.5 μm, which lays the foundation for the performance of the final device [28,35]. However, the entire film is relatively less compact, which might be caused by non-optimized spin-rate control [36]. Figure 3 shows the X-ray diffraction pattern of the CsPbBrI_2_ film. It can be found that except for the peaks of the ITO substrate marked with black dots, all samples have strong diffraction peaks at 14.4, 20.7 and 29.4°, respectively, corresponding to the (100), (110) and (200) crystal planes of CsPbBrI_2_. There are also some very weak diffraction peaks that represent a small number of impurities, such as CsPbBrI_2_, that are not completely dissolved, and these impurities have little effect on the resistance switching behavior [37,38].

**Figure 4:**
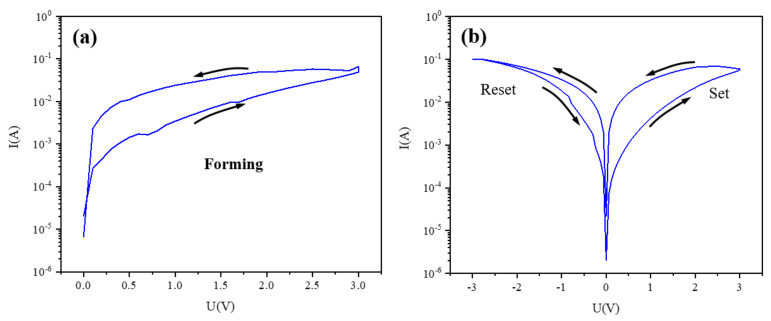
The initialization process (**a**) and typical I-V curve (**b**) of the Ag/CsPbBrI_2_/ITO device.

Next, we applied a cycle voltage of -3V-0V-3V on the Ag/CsPbBrI_2_/ITO random memory device. Figure 4a shows the initialization process of the Ag/CsPbBrI_2_/ITO resistance random-access memory device. At the initial moment, the device is in a high resistance state. After initialization, the device switches between high and low resistance states under the action of an external electric field [39,40]. The typical semi-logarithmic I-V curve shown in Figure 4a is obtained by cycling the voltage sweep in the following order 0 → −3.0 → 0 → 3.0 → 0V. As shown in Figure 4a, the device changes from a high resistance state (HRS) to a low resistance state (LRS) at the voltage of about 3 V for the first time as the voltage increases from 0V to 3V, the corresponding process is called the setting process and the corresponding voltage is called the setting voltage (Vset = 3 V). After this switching, the low resistance state (LRS) of the device remains until the negative voltage reaches -3V, then the resistive state of the device changes from low resistance state (LRS) to high resistance state (HRS), corresponding to the reset process, and the corresponding voltage is called reset voltage (Vreset = -3 V).

**Figure 5:**
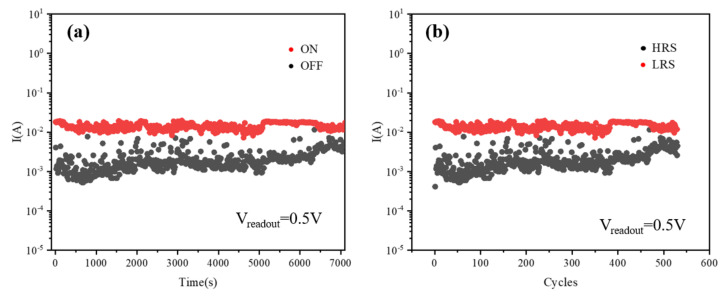
Retention test of ON/OFF current (**a**) and Switching endurance between HRS and LRS (**b**).

The memory device was found to exhibit stable current-voltage (I-V) characteristics after cycling the voltage sweep, which indicates that the device has a reversible resistance switch between the high resistance state (HRS) and the low resistance state (LRS). The retention characteristics of ON and OFF currents are obtained with a reading voltage (Vreadout) of 0.5 V for a time period of ∼10^4^ s to evaluate the non-volatile properties of the Ag/CsPbBrI_2_/ITO memory device. Figure 5 shows the retention characteristics of on-current (ON) and off-current (OFF) when the read voltage is 0.5 V. It can be seen from Figure 5a that as time increases, the current value of the device decreases slightly, but the switching ratio between the high resistance state and the low resistance state remains relatively stable, and the on-current (ON) has been able to maintain 10^4^ s, which indicates a reliable and reproducible resistive switching behavior in the Ag/CsPbBrI_2_/ITO memory device. In addition, the switching durability of the Ag/CsPbBrI_2_/ITO random memory device was also tested. As shown in Figure 5b, there is still a stable transition between resistance states and low resistance state of the Ag/CsPbBrI_2_/ITO random memory device after 500 cycles of cyclic voltage scanning, which indicates that the Ag/CsPbBrI_2_/ITO resistive random memory device based on the CsPbBrI_2_ perovskite film maintains a stable and repeatable resistance switching behavior. 

**Figure 6:**
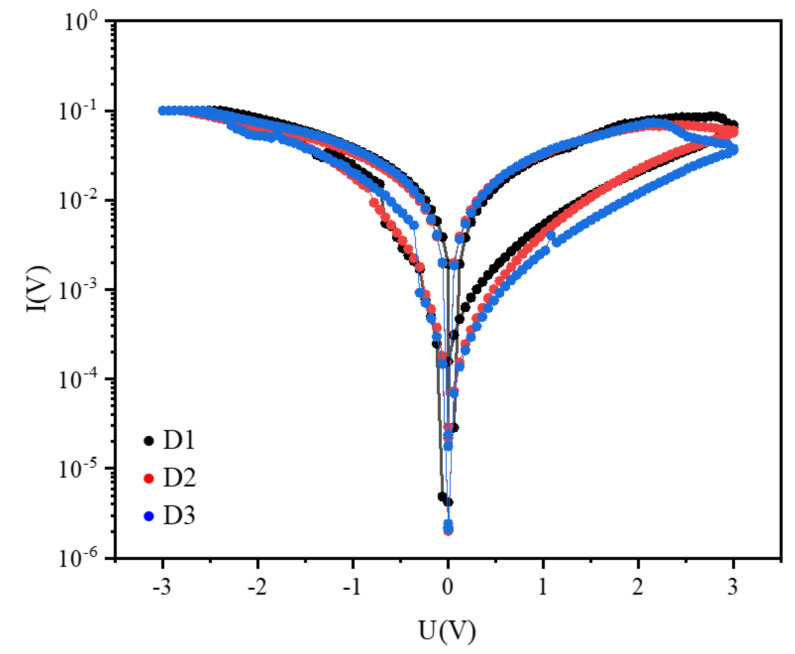
The I-V curves of different Ag/CsPbBrI_2_/ITO devices.

**Figure 7:**
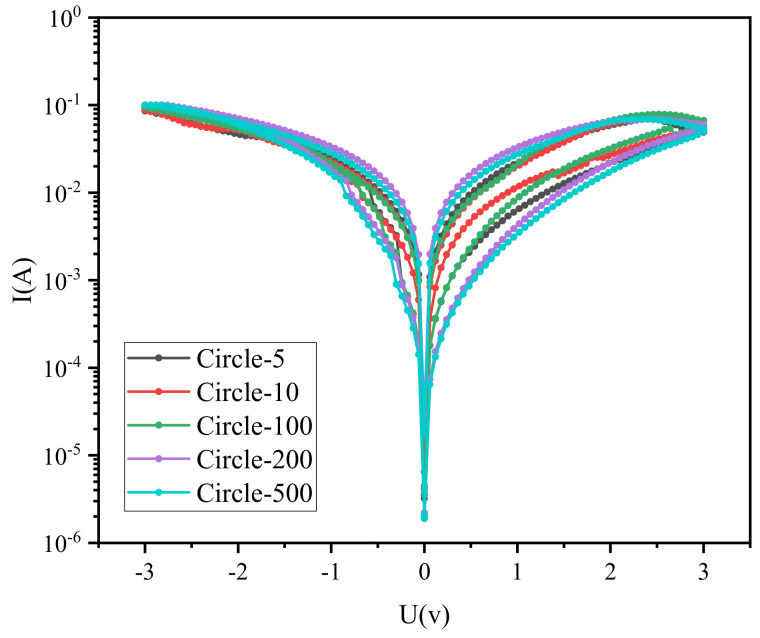
The I-V curve of Ag/CsPbBrI_2_/ITO device after different cycles of voltage sweep.

In addition, I-V curves of different batches of Ag/CsPbBrI_2_/ITO devices tests are very important for evaluating the repeatability of Ag/CsPbBrI_2_/ITO resistive random memory devices. It can be seen from Figure 6 that the resistance switching behavior of three different Ag/CsPbBrI_2_/ITO memory devices are similar after the cyclic voltage sweep. The I-V curve of Ag/CsPbBrI_2_/ITO memory device after 5, 10, 100, 200, 500 cycles is shown in Figure 7. It can be seen that the resistance switching performance of the device can still maintain a stable trend after multiple cycles of voltage scanning.

**Figure 8:**
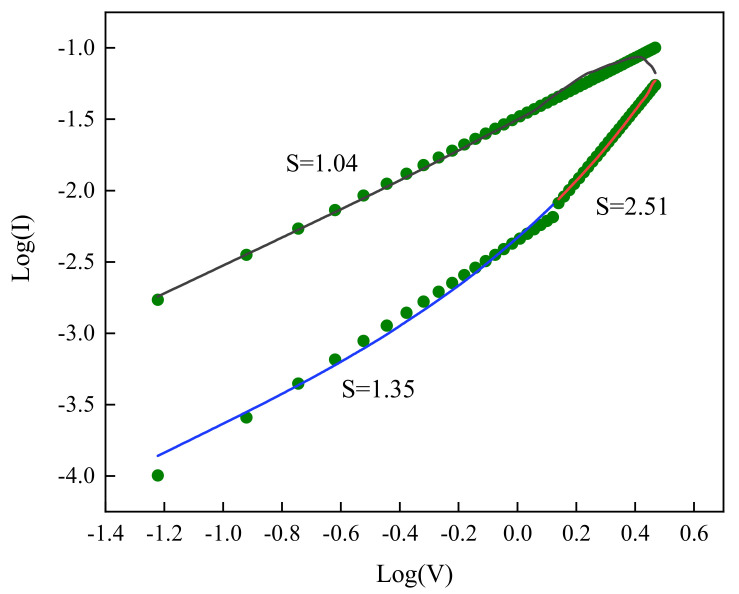
The logI-logV characteristics with fitted conduction mechanism under-voltage sweeps.

In order to further understand the resistance conversion behavior of Ag/CsPbBrI_2_/ITO resistive random memory devices, it is necessary to deeply understand the internal physical mechanism of the resistance switching effect. The previous research mainly focuses on the conductive wire model, which is generally dominated by the ohmic conduction mechanism for the conduction mechanism of the halogen perovskite resistance switching effect. Figure 8 shows the logI–logV characteristics with the positive voltage sweep. It can be seen that the slope S of the low resistance state (LRS) is 1.04, which corresponds to the ohmic conduction mechanism. According to known research reports, iodide ions and their vacancies in perovskite iodide are the dominant factors in the formation of conductive filaments, which are mainly attributed to the relatively low migration rate and relatively low migration barrier of iodide ions [41], but there is no direct experimental evidence. Although the Ag electrodes may also play a role in the formation of conductive filaments, the thicker intermediate active layer can hinder the formation of conductive metal filaments. However, the contact barrier between the upper electrode and the intermediate active layer plays an important role in the formation of conductive filaments. The sharp increase of the current during the initialization process shows the typical feature of the conductive filament model. When the device changes from a low resistance state (LRS) to a high resistance state (HRS), the slope of the curve becomes 2.51, which conforms to the spatial conduction mechanism compared with the ohmic conduction mechanism of low resistance state (LRS). With the gradual decrease of the voltage, the slope of the curve changes from 2.51 to 1.35, and the corresponding conduction mechanism of the resistance switching effect becomes an ohmic conduction mechanism. In a complete cycle, the conduction mechanism of the device has undergone the transformation of ohmic conduction–spatial conduction–ohmic conduction [42]. 

In order to fully understand the resistance switching effect and its conduction mechanism of the device, the resistance switching effect of the Ag/CsPbBrI_2_/ITO resistive random memory device is analyzed based on the conduction mechanism [28,43]. As shown in Figure 1, When a negative voltage sweep is applied to the memory device in the HRS, iodine ions can easily migrate toward the ITO electrode with the accumulation of vacancies from top to bottom electrode along the one-dimensional channel. As the negative voltage increases, the current increases sharply, corresponding to the initialization process for the vacancies to gradually migrate to form conductive filaments. The device changes from a high resistance state (HRS) to a low resistance state (LRS) and maintains a low resistance state (LRS) until the positive voltage reaches a certain value, the device begins to transition from a low resistance state (LRS) to a high resistance state (HRS). The iodide ions begin to redistribute along with the vacancies by applying a larger positive voltage leads to rupture of the conductive filaments of the vacancies, which prompts the device to switch from the high resistance state (HRS) to the low resistance state (LRS). 

**Figure 9:**
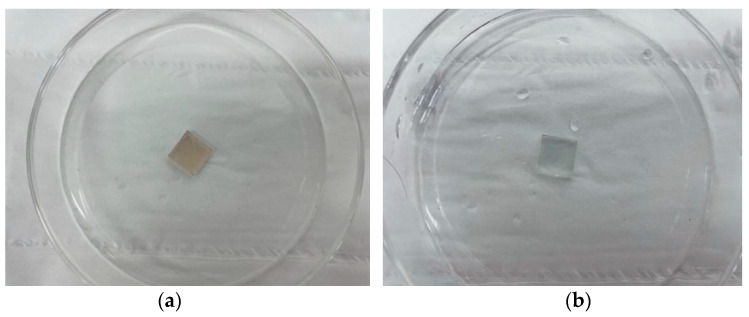
The CsPbBrI_2_/ITO film before (**a**) and after 3s (**b**) in deionized water.

In previous studies, it has been shown that some perovskite materials have transient behavior, the most representative of which is the CsPbBr_3_ perovskite film.In this study, it can be found that the film on the ITO surface quickly dissolved in the deionized water after 3s in Figure 9, which indicates that the Ag/CsPbBrI_2_/ITO resistive random memory device based on the all-inorganic halide perovskite CsPbBrI_2_ shows transient properties. In the future, this will be applied in short-period data storage unit devices, and implantable electronic devices with human or environmental sensors for the entire electronic devices can be degraded in deionized water to obtain completely self-damaging transient electronic devices [44,45].

## 4. Conclusions

In summary, the spin-coating method was used to successfully prepare CsPbBrI_2_ film, and a resistance random memory device with a sandwich structure of Ag/CsPbBrI_2_/ITO was fabricated. The surface micromorphology of CsPbBrI_2_ film and phase compositions were analyzed. The experimental results show that the Ag/CsPbI_3_/ITO memory device with high uniformity presents a non-volatile, reliable, reproducible and long-term stable resistive switching behavior of up to 500 cycles under fully open-air conditions by applying a cyclic scanning voltage to the device. The resistance switching behavior of the device is deeply analyzed by using the formation and partial fracture model of conductive filaments caused by the migration of iodide ions and the corresponding vacancies. At the same time, the transient properties of the CsPbBrI_2_ film that can quickly dissolve in deionized water make it potentially useful in short-period data storage units and implantable electronic devices with human or environmental sensors.

## Data Availability

The raw/processed data required to reproduce these findings cannot be shared at this time as the data also forms part of an ongoing study.
